# Run Charts Revisited: A Simulation Study of Run Chart Rules for Detection of Non-Random Variation in Health Care Processes

**DOI:** 10.1371/journal.pone.0113825

**Published:** 2014-11-25

**Authors:** Jacob Anhøj, Anne Vingaard Olesen

**Affiliations:** 1 Rigshospitalet, University of Copenhagen, Copenhagen, Denmark; 2 University of Aalborg, Danish Center for Healthcare Improvements, Department of Business and Management, Aalborg, Denmark; Cardiff University, United Kingdom

## Abstract

**Background:**

A run chart is a line graph of a measure plotted over time with the median as a horizontal line. The main purpose of the run chart is to identify process improvement or degradation, which may be detected by statistical tests for non-random patterns in the data sequence.

**Methods:**

We studied the sensitivity to shifts and linear drifts in simulated processes using the shift, crossings and trend rules for detecting non-random variation in run charts.

**Results:**

The shift and crossings rules are effective in detecting shifts and drifts in process centre over time while keeping the false signal rate constant around 5% and independent of the number of data points in the chart. The trend rule is virtually useless for detection of linear drift over time, the purpose it was intended for.

## Introduction

*Plotting measurements over time turns out, in my view, to be one of the most powerful devices we have for systemic learning. […] If you follow only one piece of advice from this lecture when you get home, pick a measurement you care about and begin to plot it regularly over time. You won't be sorry.*

*– Don Berwick, from his 1995 IHI Forum plenary speech "Run to Space".*

Plotting data over time is a simple method to learn from trends, patterns, and variation in data over time and to study the effect of improvement efforts.

**Figure 1:**
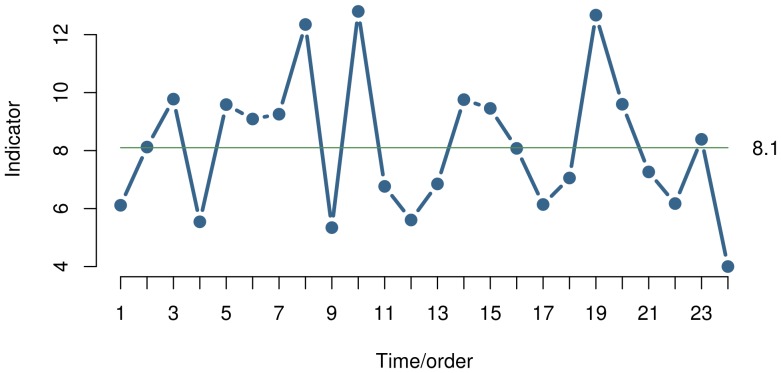
Run Chart. The run chart is a plot of a measurement over time. The horizontal line is the median, which divides the data points in two halves. The main purpose of the run chart is to identify non-random variation. For this purpose, several runs tests are available.

A run chart is a simple line graph of a measure over time with the median shown as a horizontal line dividing the data points so that half of the points are above the median and half are below (Figure 1). The main purpose of the run chart is to detect process improvement or process degradation, which will turn up as non-random patterns in the distribution of data points around the median.

If the process of interest shows only random variation, the data points will be randomly distributed around the median. “Random” meaning that we cannot know if the next data point will fall above or below the median, but that the probability of each event is 50%, and that the data points are independent. Independence means that the position of one data point does not influence the position of the next data point, that is, data are not auto-correlated. If the process shifts, these conditions are no longer true and patterns of non-random variation may be detected by statistical tests.

Thus, run charts are useful aids for improving health care processes or for detection of process degradation [1].

Runs analysis have been studied since the 1940s, and several tests have been proposed to detect non-random patterns in a data sequence.

A run is defined as a sequence of like items that is preceded and followed by one or more items of another type or by none at all. Items can be plus and minus signs, heads and tails, odd and even numbers, and numbers above and below a certain value, for example the median. Runs analysis are useful for such different purposes as digital image analysis, looking for extra-terrestrial intelligence, testing for roulette fraud and serial correlation among residuals in a regression analysis – and for detection of shifts in health care processes. Runs analysis makes no assumptions on the theoretical distribution of data.

The **runs test** was originally proposed by Swed and Eisenhart for testing the randomness of a sequence [2]. If the number of runs is too small or too large, it is an indication that the sequence is not random, e.g. positively or negatively autocorrelated. Especially, if the number is too small it will indicate that the process centre may be shifting. To do the runs test one must either do some rather complicated calculations or look up the limits for the expected number of runs in tables based on the total number of runs in the sequence and the number of items of each kind. Simplified tables for use with run charts have been developed for run charts up to 60 data points [1], [3]. For more than 60 data points, the limits can be calculated using the normal approximation of the runs distribution function [3].

Chen has proposed an alternative way of performing the runs test [4]. Instead of looking at the number of runs, Chen suggests to look at the number of shifts (crossings) in the sequence. In a run chart crossings are easily counted by counting the number of times the graph crosses the median line. The number of crossings in a random sequence will follow a binomial distribution with success probability of 0.5. Thus, the lower 5% prediction value for number of crossings is the lower 5th percentile from the cumulative distribution function of the binomial distribution with a success probability of 0.5 and n – 1 trials where n is the number of useful observations, that is, observations that do not fall on the median. The lower limit can be looked up in a statistical table or calculated using a spreadsheet or statistical software. For example, with a run chart of 20 data points, *fewer* than 6 crossings would indicate that the process is shifting.

Chen's method makes it easy to automate the calculation of signal limits. In our experience, Chen's method is also simpler to understand and teach, and counting crossings is easier for most people than counting runs.

Another widely used test for non-random variation in run charts is the **shift test**. A shift is an unusually long run of consecutive data points either above or below the median. Data points that fall on the median are not counted, that is they do not contribute to the run, nor do they break it. The cut-off value to define “unusually long” varies in the literature. Perla defines a shift as six or more consecutive data points on the same side on the median [1], while Carey and others recommend seven or eight data points depending on the purpose of the run chart and the total number of data points available [5].

The shift test is based on the theory of long runs. Schilling showed that the expected length of the longest run in a sequence depends on the total number of elements in the sequence and is predictable within limits [6]. The expected length of the longest run either above or below the median is log2(n), where n is the total number of data points not on the median (useful observations). Approximately 95% of the longest runs are predicted to be within +/− 3 of the expected value. For the purpose of detecting a change in process position, a shift, we are interested in the upper prediction limit. The upper prediction limit for longest run is log2(n)+3 rounded to the nearest integer. For example, if one has a run chart of 20 data points, the expected longest run would be 4, and a run of *more* than 7 data points would indicate a shift in process level.

The **trend test** is also commonly used. A trend is an unusually long run of data points all going up or down. Conceptually, a trend is merely a special form of a run, where like items are defined as data points that are bigger or smaller than the preceding one. The trend test was developed by Olmstead who provided tables and formulas for the probabilities of trends of different lengths depending on the total number of data points [7]. For example, with less than 27 data points in total, the chance of having a trend of 6 or more data points going up or down is less than 5%. Note that Olmstead defines a trend as the number of jumps rather than the number of data points that surround the jumps (which, of cause, is one more than the number of jumps).

Although the trend rule has been shown to be ineffective [8], it is still widely used in health care.

Willemain has suggested a type of runs analysis using **level crossings**, which is a metric based on lengths of adjacent runs. However, this method is only appropriate in data rich environments with, say, thousands of observations per day, which is rarely the case in health care [9].

In addition to the above mentioned rules, it is recommended also to look for other “unnatural” patterns in data that may indicate the presence of non-random variation. These patterns may include cyclic behaviour or obviously different data points.

The aim of this study was to propose a set of rules that applied together will detect process improvement or degradation as soon as possible while having a low rate of false signals when only random variation is present.

## Methods

We studied the signal rates in simulated run charts with 2 to 100 data points under influence of linear drifts and non-linear shifts in the sample distribution mean.

We wrote the simulation program in the R programming language (v. 3.1.1) and used some functions from the add-on package reshape2 (v. 1.2.4) for data management and functions from the lattice package (v. 0.20–29) for graphs [10]. The programme is available for download as supplementary material to this article (Material S1).

For the study on the effect of shifts in process mean, the program did 1000 simulations of each run chart of length 2 to 100 with samples from a normal distribution with a standard deviation of 1 and distribution mean of 0.0, 0.5, 1.0, 1.5, and 2.0 respectively. In addition, the run charts with sample mean of zero was analysed with both a fixed median of 0 and with a floating median defined as the actual median of the numbers in the run chart. All other analyses were done with a fixed median of 0.

To study the effect of linear drift in process mean, the program did 1000 simulations of each run chart of length 2 to 100 while introducing an increase of 0.0, 0.1, 0.2, and 0.3 respectively in distribution mean between consecutive sample data points.

We studied the following rules for detection of non-random variation:

Shift rule: A shift is present if any run of consecutive data points on the same side of the median is longer than the prediction limit.

Crossings rule: A crossings signal is present if the number of times the graph crosses the median is smaller than the prediction limit.

Trend rule: A trend is present if the longest run of data points going up or down is longer than the prediction limit.

Prediction limits for shifts and crossings in run charts with 10 to 100 data points are tabulated in table 1. A trend was signalled if the p-value calculated using formula 6 in Olmstead's paper was less than 0.05 [7].

**Table 1:** Prediction limits for longest run and number of crossings.

Number of useful observations (n)	Upper prediction limit for longest run	Lower prediction limit for number of crossings
	= round(log2(n)+3)	= qbinom(0.05, n–1, 0.5)
10	6	2
11	6	2
12	7	3
13	7	3
14	7	4
15	7	4
16	7	4
17	7	5
18	7	5
19	7	6
20	7	6
21	7	6
22	7	7
23	8	7
24	8	8
25	8	8
26	8	8
27	8	9
28	8	9
29	8	10
30	8	10
31	8	11
32	8	11
33	8	11
34	8	12
35	8	12
36	8	13
37	8	13
38	8	14
39	8	14
40	8	14
41	8	15
42	8	15
43	8	16
44	8	16
45	8	17
46	9	17
47	9	17
48	9	18
49	9	18
50	9	19
51	9	19
52	9	20
53	9	20
54	9	21
55	9	21
56	9	21
57	9	22
58	9	22
59	9	23
60	9	23
61	9	24
62	9	24
63	9	25
64	9	25
65	9	25
66	9	26
67	9	26
68	9	27
69	9	27
70	9	28
71	9	28
72	9	29
73	9	29
74	9	29
75	9	30
76	9	30
77	9	31
78	9	31
79	9	32
80	9	32
81	9	33
82	9	33
83	9	34
84	9	34
85	9	34
86	9	35
87	9	35
88	9	36
89	9	36
90	9	37
91	10	37
92	10	38
93	10	38
94	10	39
95	10	39
96	10	39
97	10	40
98	10	40
99	10	41
100	10	41

Use the table to identify signals of non-random variation in run charts by comparing the longest run and the number of crossings to the prediction limits in the table. The number of useful observations is the number of data points that are not on the median.

Non-random variation is present if the longest run is *longer* than the prediction limit *or* if the number of crossings is *less* than the prediction limit.

The prediction limits may be calculated in R using the provided formulas.

For comparison, we also studied the **three-sigma rule**, which is used with Shewhart control charts. The three-sigma signal is triggered when any data point is further than 3 standard deviations away from the overall process average [11]. Since simulation data were created from a standard normal distribution with a known mean of 0 and a standard deviation of 1, we used +/− 3 as sigma limits.

The outcome from each simulation was the proportion of run charts which triggered the rule, that is, the simulated power of the test with respect to detection of the shift under study.

## Results

**Figure 2:**
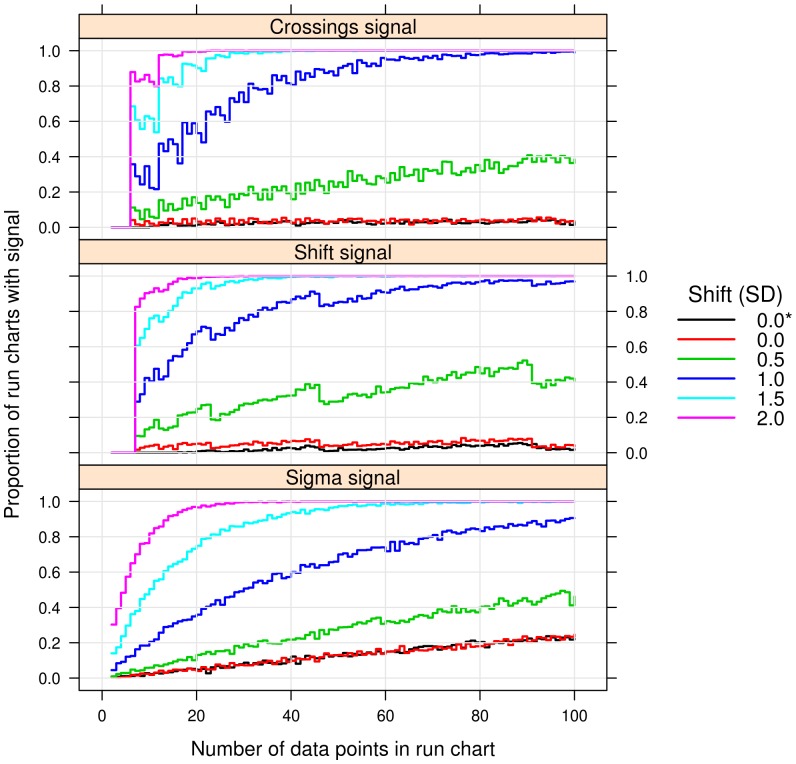
Signal rates during shift in process mean. Each panel shows the proportion of simulated run charts (N = 1000) that signaled the runs test in run charts of lengths from 2 to 100. Each line shows the signal rate when using random samples from a normal distribution with a standard deviation of 1 and a mean of 0, 0.5, 1, 1,5, and 2 respectively. *  =  floating median. Other lines used a fixed median of 0.

Figure 2 shows that when no shift is present in the data, the (false) signal rates for the shift and crossings rules suggested in this paper are approximately 5% and independent of the number of data points in the run chart. The (true) signal rates increase when shifts are introduced in the data. The larger the shift, the quicker the run charts pick up the signal.

For example, when a shift in process mean of 1 standard deviation is present, approximately 70% and 60% of run charts with 20 data points will signal the shift and crossings rules respectively. If the shift is more than 1.5 standard deviation, over 90% of run charts with 20 data points detect the change.

The shift rule has a slightly higher signal rate with a fixed median compared to a floating median. The difference levels out as the number of data points increases.

When combining the shift and crossings rules so that a signal is considered present if either the shift or the crossings rule is triggered, the sensitivity to shifts in data increased at the cost of a slightly elevated false signal rate (results not shown).

The three-sigma rule is less sensitive to shifts up to 2 standard deviations than the shift and crossings rules and shows an increasing false signal rate as the number of data points increases. At 40 data points the false signal rate is around 10%. It should be noted that, in practice, it is common to adjust the sigma limits to the number of data points to achieve a fixed false signal rate.

**Figure 3:**
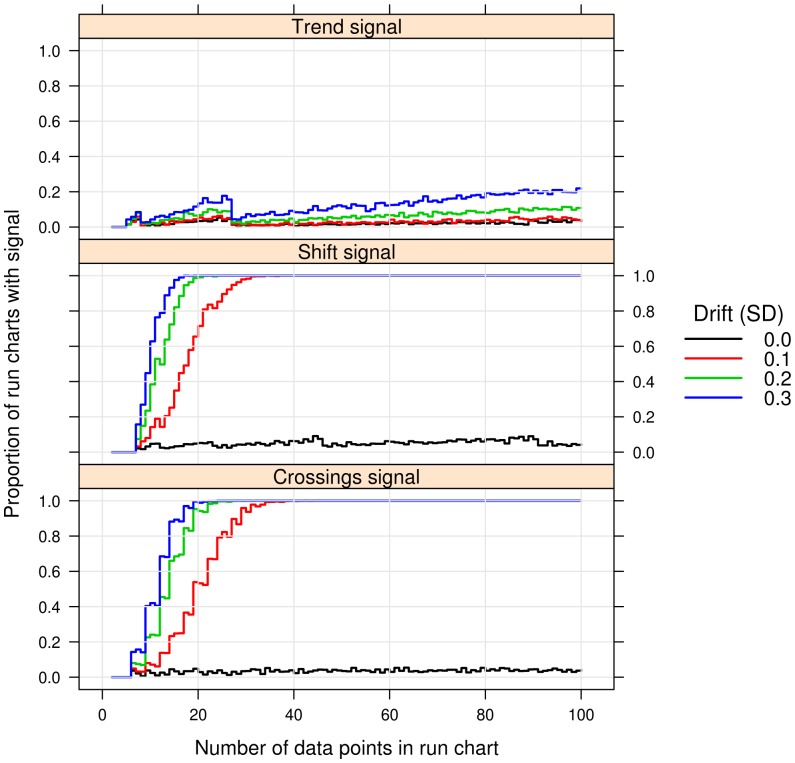
Signal rates during linear drift in process mean. Each panel shows the proportion of simulated run charts (N = 1000) that signaled the runs test in run charts of different lengths from 2 to 100. Each line shows the signal rate when using random samples from a normal distribution with a standard deviation of 1 while introducing a drift in sample mean of 0, 0.1, 0.2, and 0.3 respectively between sample data points.

Figure 3 shows that even when a drift of 0.3 standard deviation is added to each sample data point, only about 20% of run charts with 100 data points will signal the trend rule. In contrast, the shift and crossings rules pick up the drift with almost 100% certainty already at 20 data points. Thus, the trend rule is virtually useless for detection of linear drift over time, the purpose it was intended for.

## Discussion

This simulation study shows that the suggested shift and crossings rules are effective in detecting moderate to large shifts and drifts in process level over time while keeping the false signal rate constant around 5% and independent of the number of data points in the chart.

The trend rule is performing so poorly compared to the other rules, that we question its appropriateness.

To our knowledge, this study is the first to study the performance of run charts under simulated shifts and drift in process mean.

Traditionally, the performance of Shewhart control charts is characterized by the average run length (ARL) to signal. ARL is the average number of data points it takes to pick up a change in process level. For practical and didactical reasons we chose to plot signal rates as a function of run chart length as our metric. First, it turned out to be impractical to identify ARL for our run chart rules when no signals were present. Because the prediction limits are dynamically adapted to the number of data points these simulations often entered “endless” loops creating extremely long data sequences making the simulation programme run for several days without completion. Second, the graphical presentation of signal rates are, in our experience, more intuitively understandable for clinicians without specific statistical training than are ARL's.

It may be argued that the simulations used in our study are for a fixed number of observations, while in practice one will apply the run chart rules sequentially, deciding after each data point if non-random variation is present. However, it is our experience from using these rules in practice for several years on real life data that the rules work well in practice and offer good protection against false positive signals and are effective in detecting “real” change in process performance.

The shift rule proposed in this study is more conservative than the shift rules that are usually suggested [1], [3], [5]. Perla and Provost suggest using only 6 data points to signal a shift. Carey suggests 7 data points for a run chart with up to 19 data points. The shift rule we suggest takes at least 8 data points to signal a shift if one has between 12 and 22 data points in total. Of course, the choice of cut off value is a trade-off between the need to quickly pick up a signal when the process is changing and the risk of picking up false signals when no change is present. One should be aware that relaxing the rules will result in higher false signal rates.

Run charts have several advantages over control charts. First, run charts are easier to construct and teach. Pen and paper is all it takes. Second, run charts make no assumptions about the theoretical distribution of data, which make them especially useful when baseline data are not available to characterise the process prior to a control chart analysis. Third, when using the shift and crossings rules proposed in this study rather than the usual fixed signal limits, the false signal rate is independent of the number of data points. Finally, as our results show, the run chart rules we propose have higher sensitivity and specificity to moderate (∼1.5 SD) changes in process performance than the sigma rule alone.

As a consequence, we suggest always studying process data with run charts before applying control charts. If a run chart identifies non-random variation, it could mean many things including a lack of independence in the data, which would invalidate the control chart.

However, having no control limits, run charts are not capable of determining if any single data point deviates significantly from the rest. Thus run charts will not detect large and transient shifts and may be slower than control charts to detect large (>2 SD) permanent shifts in process mean. Also, run charts are unable to determine if the process is statistically stable [1]. But even if stability may be the end goal of all improvement work, induced instability as a result of ongoing improvement is the short term goal.

**Figure 4:**
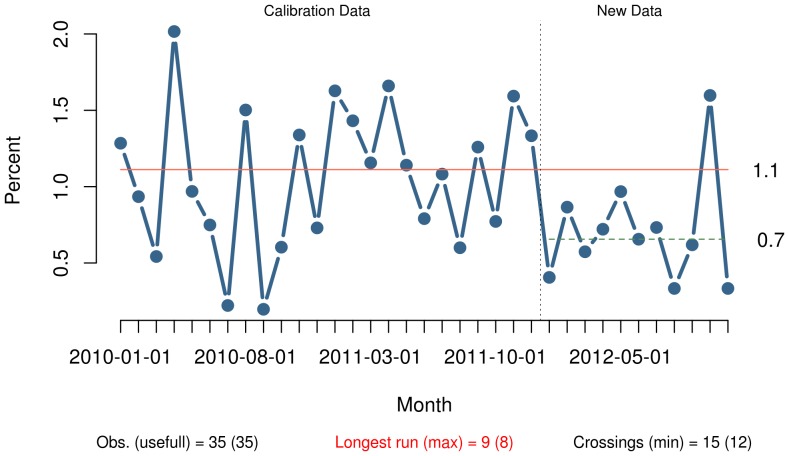
Post-op mortality. This example illustrates the principles suggested in this article used on data from a hospital that reduced the post-operative mortality after implementing the WHO Safe Surgery Checklist. The median is based on the first 24 data points. After the baseline period a shift appears suggesting a reduction in post-op mortality from 1.1% to 0.7%. The shift is verified by a run of 9 data points below the median. (Unpublished data used with permission).

The important thing to consider before choosing the right chart is the purpose of the chart. In our experience, run charts are especially useful for determining if improvement is happening when improvement work is ongoing and baseline data are not available. In these situations, the use of control charts may, as mentioned, be inappropriate. In the last section we suggest some guidelines for the use of run charts for improvement work, and a practical example is shown in Figure 4.

Control charts, on the other hand, are especially useful for continuous optimization (reducing variation) and control of processes which already have been brought to acceptable levels of performance. Some types of control charts like CUSUM and EWMA charts are very sensitive to small shifts in process mean over time. These charts are useful for continuous monitoring of critical outcome measures that have been optimized and stabilised in advance in order to detect process degradation as quickly as possible. Mohammed provides several useful examples on the construction and use of control charts in healthcare [12].

For basic Shewhart control charts, we suggest combining the sigma test with the runs and crossings tests using the mean as reference rather than using a fixed limit for longest run, which would be the traditional approach.

When using a fixed median, as in our simulation programme, and in the example in Figure 4, we assume that the median is established from baseline data before any improvement initiative is undertaken. This is, of course, the optimal situation. Baseline data may provide important information on trends and patterns, e.g. autocorrelation, already present in the process, which may be useful in guiding the improvement work [13].

However, getting baseline data beforehand is not always possible in reality. Often improvement programmes are initiated without available baseline data, and waiting for baseline data may even be unethical. In those situations, measurements start together with the improvement initiative. This is not necessarily a problem, as any significant effect will show itself as non-random variation relatively quickly, as demonstrated in the simulation study. Perla provides useful guidance on sampling considerations in ongoing improvement work [14].

In conclusion: This study shows that the use of the shift and crossings rules on run charts is effective for detection of non-random variation due to shifts and drifts in process mean while keeping the false signal rate at an acceptable level. We recommend that the use of the trend rule should be discontinued.

## Guidelines for Using and Interpreting Run Charts for Health Care Improvement

1. Define the measure of interest and, if relevant, the target.
2. Collect data and plot the measure in time order. Connect the data points with straight lines.
3. After at least 12, preferably 20 or more, data points, add a horizontal line representing the median, that is half the data points are above and half are below the median.
4. Count the number of useful observations, that is, data points not on the median.
5. Find the longest run of consecutive data points above or below the median. Data points that fall on the median should be disregarded. They do not contribute to the run, nor do they break the run.
6. Count the number of times the line crosses the median.
7. Compare the longest run and the number of crossings with the predictions limits in table 1. Non-random variation is present if the longest run is *longer* than the prediction limit *or* if the number of crossings is *less* than the prediction limit.
8. Look for other patterns in data that may signal non-random variation, for example obvious outliers or cycles caused by seasonal or diurnal changes in process performance. Be careful though, not to confuse random high or low data points with true outliers. If in doubt, use a control chart.
9. If only random variation is present after at least 20 data points, lock the median and continue to collect and plot data while improving the process. In case of unwanted non-random variation, identify and eliminate the cause(s).
10. If, or when, the target has been reached, calculate the new median or define the target as the new median.
11. Improvement has been sustained when the process shows only random variation at a better level.
12. Consider using a control chart to monitor the process onwards.

Figure 4 illustrates the use of this guideline on data from a hospital that reduced the post-operative mortality after implementing the WHO Safe Surgery Checklist.

## Supporting Information

Material S1 **The simulation programme will run on an installation of R with the add-on packages lattice and reshape2.** Instructions are included in the programme file.
(R) Click here for additional data file.
